# Genome-wide survey of tandem repeats by nanopore sequencing shows that disease-associated repeats are more polymorphic in the general population

**DOI:** 10.1186/s12920-020-00853-3

**Published:** 2021-01-07

**Authors:** Satomi Mitsuhashi, Martin C. Frith, Naomichi Matsumoto

**Affiliations:** 1 Department of Human Genetics, Yokohama City University Graduate School of Medicine, Fukuura 3-9, Kanazawa-ku, Yokohama, 236-0004 Japan; 2 Department of Genomic Function and Diversity, Medical Research Institute, Tokyo Medical and Dental University, M&D Tower 24F, 1-5-45 Yushima, Bunkyo-ku, Tokyo, 113-8510 Japan; 3 Artificial Intelligence Research Center, National Institute of Advanced Industrial Science and Technology (AIST), Tokyo, Japan; 4 Graduate School of Frontier Sciences, University of Tokyo, Chiba, Japan; 5 Computational Bio Big-Data Open Innovation Laboratory (CBBD-OIL), AIST, Tokyo, Japan

**Keywords:** Nanopore long read sequencing, Tandem repeats, Triplet repeat disease, Genome-wide analysis

## Abstract

**Background:**

Tandem repeats are highly mutable and contribute to the development of human disease by a variety of mechanisms. It is difficult to predict which tandem repeats may cause a disease. One hypothesis is that changeable tandem repeats are the source of genetic diseases, because disease-causing repeats are polymorphic in healthy individuals. However, it is not clear whether disease-causing repeats are more polymorphic than other repeats.

**Methods:**

We performed a genome-wide survey of the millions of human tandem repeats using publicly available long read genome sequencing data from 21 humans. We measured tandem repeat copy number changes using tandem-genotypes. Length variation of known disease-associated repeats was compared to other repeat loci.

**Results:**

We found that known Mendelian disease-causing or disease-associated repeats, especially CAG and 5′UTR GGC repeats, are relatively long and polymorphic in the general population. We also show that repeat lengths of two disease-causing tandem repeats, in *ATXN3* and *GLS*, are correlated with near-by GWAS SNP genotypes.

**Conclusions:**

We provide a catalog of polymorphic tandem repeats across a variety of repeat unit lengths and sequences, from long read sequencing data. This method especially if used in genome wide association study, may indicate possible new candidates of pathogenic or biologically important tandem repeats in human genomes.

## Background

There are more than 30 rare Mendelian diseases caused by tandem repeat expansions in human genomes [1]. Genome-wide surveys of tandem repeats in individual genomes are now feasible due to the development of high-throughput sequencing technologies, which enable direct identification of large pathogenic expansions [2–4]. However, it is still difficult to predict which tandem repeats cause disease, because there are thousands of tandem repeats in each individual that are different from the reference genome. Usually pathogenic expansions are + 100 to ~ 10,000 base-pairs, and the risk cutoff is beyond ~ 100 base-pairs [1, 2]. Some disease-causing repeats are polymorphic even in healthy individuals [5]. If disease-causing tandem repeats have distinct variation in the general population, compared to other repeats, that would help identify novel disease-causing repeat candidates.

Although tandem repeats are highly mutable and can affect phenotype, they are rarely considered in genome-wide association studies (GWAS). GWAS has found many polymorphisms that have significant but weak association with phenotypes, so far failing usually to give satisfying genetic explanations of the phenotypes. As tandem repeats’ rapid evolution causes them to have weak association with nearby polymorphisms, we may hypothesize that repeats explain these phenotypes, as represented in previous studies [6, 7].

Current genome-wide studies of tandem repeats using short read sequencers are mainly focusing on short repeats (repeat unit range: 1–6 bp) [8] due to the limitation of detecting long repeats. Current long read sequencing technologies (PacBio and Nanopore) have achieved reads longer than 10 kb on average, which have a high chance to cover whole tandem repeats including flanking unique sequences [9, 10]. However, to the best of our knowledge, there has been no study that characterizes the genotypic variation of disease-causing and other tandem repeats using only long reads.

Until recently, most of the known disease-causing tandem repeats are CAG or GGC triplet repeats [1], although there are a few exceptions; quadruplet repeat (CCTG) in Myotonic Dystrophy type 2 (MIM#602668), and sextuplet repeat (GGGGCC) in Frontotemporal dementia and/or amyotrophic lateral sclerosis (ALS) (MIM#614260). CAG and GGC triplet diseases have three major disease mechanisms: poly-glutamine diseases (CAG), poly-alanine diseases (GGC), or 5′UTR GGC expansion diseases [11–13]. In addition to triplet repeats, pathogenic expansions of quintuplet repeat loci (represented as AAAAT in hg38) are associated with myoclonic epilepsies. In 2018 and 2019, six AAAAT repeat loci were reported [4, 14–16] in addition to *BEAN1* which causes spinocerebellar ataxia 31 (MIM#117210) [17]. We focus on these triplet and quintuplet repeats so that we can test several disease loci.

Our recently developed tool, tandem-genotypes, can robustly detect tandem repeat changes from whole genome long read sequencing data [18]. Here, we used this tool to measure tandem repeats in publicly available nanopore long read whole genome sequencing data. We show that certain types of disease-causing tandem repeats have greater length variation than other repeats.

## Methods

### Long read sequencing and mapping to the reference genome

We used 21 long read whole genome sequencing datasets, from 21 humans (Additional file 1: Table S1). Fifteen of these are from previous studies [10, 19, 20]. The other six were sequenced by our group, using Nanopore PromethION as previously described [3], with DNA obtained from lymphoblastoid cell lines from the Coriell Institute Cell Repository (coriell.org). Reads were mapped to the human reference genome GRCh38 using LAST according to the instructions (https://github.com/mcfrith/last-rna/blob/master/last-long-reads.md), with repeat-masked reference genome.

last-train GRCh38 data.fa > train-out

lastal -p train-out GRCh38 data.fa | last-split > alignment.maf

### Tandem repeat detection

Tandem repeats in the human reference genome GRCh38 were detected using tantan (http://cbrc3.cbrc.jp/~martin/tantan/) [21], with this command:

tantan -f4 -w2000 GRCh38.fa > tantan-out

### Prediction of tandem repeat copy number changes relative to the reference

Tandem-repeat copy number changes relative to the reference were predicted using tandem-genotypes. We used one non-default parameter, n = 10 instead of n = 60, to make it more specific but less sensitive. This is because the precise boundaries of (inexact) repeats are ambiguous: n = 10 makes it less likely to regard an insertion near a repeat as an expansion of the repeat, but more likely to miss expansions of repeats with fuzzy boundaries [18]. Disease-associated tandem repeats were analyzed separately, using the repeat annotations in Table 1.

**Table 1:** Variability of triplet- and quintuplet-repeat disease locus in 21 individuals detected by long read nanopore sequencing. OMIM: Online Mendelian Inheritance in Men

Chromosome	Start	End	Repeat	Gene	Function	Repeat copy number change from the reference					Absolute repeat length			Disease	OMIM
						Mean	Median	IQR	Q1	Q3	Mean length (bp)	Q1 length (bp)	Q3 length (bp)		
chr2	176,093,058	176,093,103	GGC	HOXD13	Coding	− 0.1	0	1.00	− 1.00	0.00	45	42	45	Syndactyly, type V	186,300
chr4	41,745,971	41,746,031	GGC	PHOX2B	Coding	0.0	0	2.00	− 1.00	1.00	60	57	63	Congenital central hypoventilation syndrome	209,880
chr6	45,422,750	45,422,801	GGC	RUNX2	Coding	− 0.7	0	1.00	− 1.00	0.00	49	48	51	Cleidocranial dysplasia, forme fruste, with brachydactyly	119,600
chr7	27,199,924	27,199,966	GGC	HOXA13	Coding	0.0	0	2.00	− 1.00	1.00	42	39	45	Hand-foot-genital syndrome	140,000
chr13	99,985,448	99,985,493	GGC	ZIC2	Coding	0.1	0	2.00	− 1.00	1.00	45	42	48	Holoprosencephaly	609,637
chr14	23,321,472	23,321,502	GGC	PABPN1	Coding	− 0.1	0	1.00	− 1.00	0.00	30	27	30	Oculopharyngeal muscular dystrophy	164,300
chrX	25,013,649	25,013,697	GGC	ARX	Coding	0.2	0	1.00	0.00	1.00	49	48	51	Early infantile epileptic encephalopathy	308,350
chrX	140,504,316	140,504,361	GGC	SOX3	Coding	0.0	0	2.00	− 1.00	1.00	45	42	48	Mental retardation with isolated growth hormone deficiency	300,123
chr1	149,390,802	149,390,842	GGC	NOTCH2NLC	5′UTR	6.7	6	5.00	3.00	8.00	60	49	64	Neuronal intranuclear inclusion disease	603,472
chr8	104,588,965	104,588,999	CCG	LRP12	5′UTR	0.3	0	2.00	− 1.00	1.00	35	31	37	Ocuropharyngodistal myopathy	164,310
chr10	79,826,315	79,826,404	GGC	LOC642361,NUTM2B-AS1	Non coding exon	2.6	3	3.00	1.00	4.00	97	92	101	Ocuropharyngodistal myopathy	618,637
chr19	14,496,042	14,496,085	CCG	GIPC1	5′UTR	1.6	0	3.00	− 1.00	2.00	48	40	49	Ocuropharyngodistal myopathy	618,940
chrX	147,912,050	147,912,110	GGC	FMR1	5′UTR	8.7	9	5.00	6.00	11.00	86	78	93	Fragile X syndrome/tremor-ataxia syndrome	300,624/300,623
chrX	148,500,637	148,500,682	GGC	AFF2	5′UTR	2.2	2	5.00	0.00	5.00	52	45	60	Fragile X syndrome	309,548
chr3	63,912,685	63,912,715	CAG	ATXN7	Coding	0.2	0	2.00	− 1.00	1.00	30	27	33	Spinocerebellar ataxia 7	164,500
chr4	3,074,876	3,074,939	CAG	HTT	Coding	− 1.2	− 2	3.00	− 3.00	0.00	59	54	63	Huntington disease	143,100
chr6	16,327,635	16,327,722	CAG	ATXN1	Coding	− 1.1	− 1	4.00	− 3.00	1.00	84	78	90	Spinocerebellar ataxia 1	164,400
chr6	170,561,907	170,562,021	CAG	TBP	Coding	− 2.7	− 3	3.00	− 4.00	− 1.00	106	102	111	Spinocerebellar ataxia 17	607,136
chr12	6,936,716	6,936,773	CAG	ATN1	Coding	− 2.5	− 2	5.00	− 5.00	0.00	49	42	57	Dentatorubral-pallidoluysian atrophy	125,370
chr12	111,598,950	111,599,019	CAG	ATXN2	Coding	− 1.4	− 1	1.00	− 2.00	− 1.00	65	63	66	Spinocerebellar ataxia 2	183,090
chr14	92,071,010	92,071,040	CAG	ATXN3	Coding	7.2	8	10.00	1.00	11.00	51	33	63	Spinocerebellar ataxia 3	109,150
chr19	13,207,858	13,207,897	CAG	CACNA1A	Coding	− 2.0	− 2	2.00	− 3.00	-1.00	33	30	36	Spinocerebellar ataxia 6	183,086
chrX	67,545,317	67,545,386	CAG	AR	Coding	− 1.7	− 2	5.00	− 4.00	1.00	64	57	72	Spinal and Bulbar Muscular Atrophy	313,200
chr2	190,880,868	190,880,920	GCA	GLS	5′UTR	− 4.3	− 3	6.75	− 8.00	− 1.25	39	28	48	Global developmental delay, progressive ataxia, and elevated glutamine	618,412
chr18	55,586,153	55,586,229	AGC	TCF4	5′UTR	− 5.5	− 7	9.00	− 10.00	− 1.00	59	46	73	Fuchs corneal dystrophy	602,272
chr19	45,770,204	45,770,264	CAG	DMPK	3′UTR	− 9.4	− 9	5.00	− 12.00	− 7.00	32	24	39	Myotonic dystrophy 1	160,900
chr2	96,197,066	96,197,124	AAAAT	STARD7	intron	2.9	1	3.00	0.00	3.00	72	58	73	Myoclonic epilepsy	607,876
chr3	183,712,187	183,712,226	TTTTA	YEATS2	intron	11.0	2	30.00	0.00	30.00	94	39	189	Myoclonic epilepsy	615,127
chr4	159,342,526	159,342,618	AAAAT	RAPGEF2	Intron	0.5	1	1.00	0.00	1.00	94	92	97	Epilepsy, familial adult myoclonic, 7	618,075
chr5	10,356,339	10,356,411	AAAAT	MARCHF6	Intron	0.4	0	1.00	0.00	1.00	74	72	77	Myoclonic epilepsy	613,608
chr8	118,366,815	118,366,918	AAAAT	SAMD12	Intron	− 0.8	− 1	3.00	− 2.00	1.00	99	93	108	Epilepsy, familial adult myoclonic, 1	601,068
chr16	24,613,438	24,613,532	AAAAT	TNRC6A	Intron	− 5.0	− 5	2.00	− 6.00	− 4.00	69	64	74	Epilepsy, familial adult myoclonic, 6	618,074
chr16	66,490,396	66,490,466	AAAAT	BEAN1	Intron	24.1	3	7.00	− 1.00	6.00	190	65	100	Spinocerebellar ataxia 31	117,210

tandem-genotypes -n10 -g refFlat.txt tantan-out alignnment.maf > out

All tandem-genotypes output files from 21 datasets were merged like this:

tandem-genotypes-join file1 file2 file3… > merged-file

IQR and mean length were calculated from tandem-genotypes output using GNU datamash (https://www.gnu.org/software/datamash/).

### Repeat disease selection

We selected triplet-repeat and quintuplet-repeat diseases, because several diseases are known in this category. We took these repeats from a previously published article [1], and recently discovered repeat diseases were added by manual literature search.

### Phasing the repeat and near-by GWAS SNP

Phasing of a disease-associated (*ATXN3* or *GLS*) tandem-repeat and nearby GWAS SNP (< 10 kb) [22] was done from consensus sequences of the DNA reads. Briefly, a repeat’s copy number in each of the two alleles was estimated by tandem-genotypes, then the reads from the two alleles were merged into two consensus sequences, and re-aligned to the reference genome. tandem-genotypes-merge merges these reads using lamassemble [23, 24]:

tandem-genotypes -o2 -v repeat-locus alignnment.maf > out

tandem-genotypes-merge reads.fa train-out out > merged.fa

## Results

We identified tandem repeats in a human reference genome (GRCh38) using tantan [21] (http://cbrc3.cbrc.jp/~martin/tantan/). In total, 3,347,418 loci were identified, with the repeat units ranging from 1 to 2000 bp. We used 21 publicly available long read whole genome sequencing datasets (we suppose they do not have pathogenic tandem repeat expansions), with average coverage of 27x (ranging 8x–48x, Additional file 1: Table S1). tandem-genotypes predicted lengths for more than 98% of the 3 million tandem repeats (Additional file 1: Table S1), including 215,561 triplet repeats.

We investigated 12 CAG and 14 GGC triplet repeat and 7 AAATA quintuplet repeat disease loci (Table 1), and plotted the distribution of copy number changes from the reference in all the reads. We found that disease-causing repeats show different distribution from other non-disease repeats (Additional file 2: Fig S1A–C). We randomly extracted the same number of non-disease repeat loci for comparison to the disease repeat loci (CAG: n = 12, GGC: n = 14, AAAAT: n = 7) (Additional file 2: Figure S1). This supports our hypothesis that disease-causing tandem repeats are more polymorphic among the normal population than other loci.

Given that different repeat sequences may have different mutation rates [25], we compared the ten kinds of non-disease triplet repeats (All triplet repeats can be categorized into 10 kinds. Note that AAC repeats includes AAC, ACA, CAA, GTT, TGT, TTG repeats) (Additional file 2: Figure S2). We plotted the variation of repeat length (interquartile range (IQR) of repeat-unit count from each read), and mean repeat length, at each exonic locus (including UTR). Most of the non-disease triplet repeats have little or no length polymorphism. A large fraction (> 94% of all repeats) have IQR 2 or less, while disease causing tandem repeats usually show more variation (always more than 2) (Table 1). It is of interest that GGC and CAG repeats have more polymorphic loci than other repeat structures (Additional file 2: Figure S2). In addition, shorter-unit repeats are more numerous and more variable (Additional file 2: Figure S3 A, B). Therefore, we analyzed the variation (IQR) and repeat length for disease causing repeats in comparison to other repeats considering the repeat unit and repeat location.

**Fig. 1:**
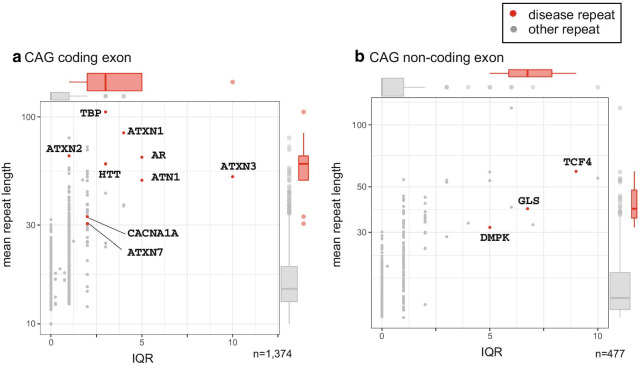
Variation (IQR, interquartile range) and length of repeats with disease-associated sequences. coding CAG repeats (**a**), and non-coding exonic CAG repeats (**b**). x-axis: IQR, y-axis: mean repeat length (bp). n provides the numbers of repeat loci. In merged boxplots on the right and upper, ranges are the 25th and 75th percentiles, dots are outliers and lines in boxes are median

**Fig. 2:**
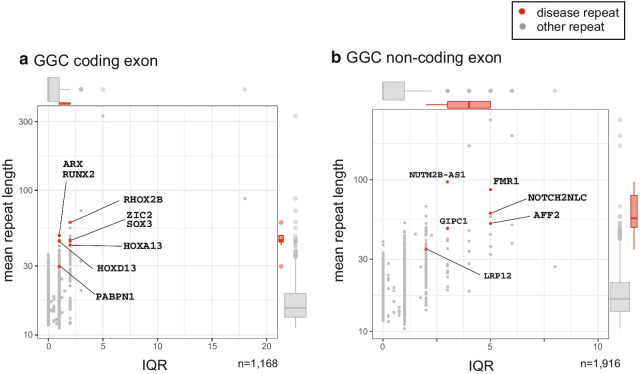
Variation (IQR) and length of repeats with disease-associated sequences. coding GGC repeats (**a**), and non-coding exonic GGC repeats (**b**). x-axis: IQR, y-axis: mean repeat length (bp). n provides the numbers of repeat loci. In merged boxplots on the right and upper, ranges are the 25th and 75th percentiles, dots are outliers and lines in boxes are median

**Fig. 3:**
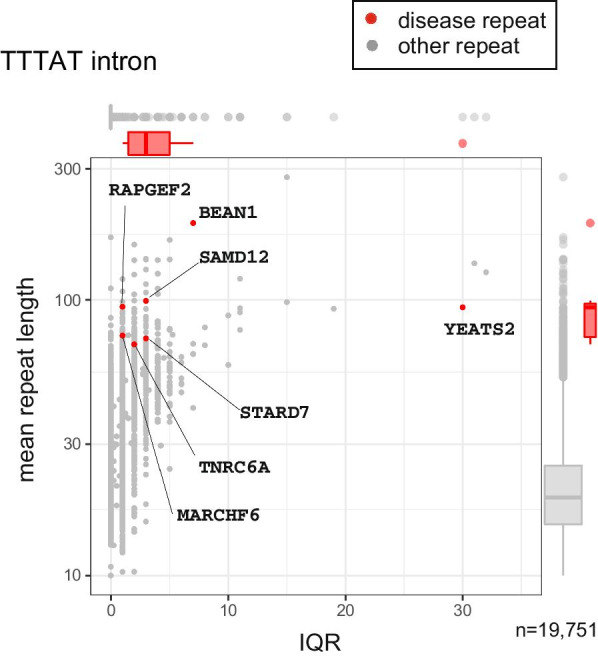
Variation (IQR) and length of repeats with disease-associated intronic AAAAT sequences. x-axis: IQR, y-axis: mean repeat length (bp). n provides the numbers of repeat loci. In merged boxplots on the right and upper, ranges are the 25th and 75th percentiles, dots are outliers and lines in boxes are median

Disease-associating CAG repeats are longer and more variable than most other CAG repeats (Fig. 1a, b, Table 1). We showed coding and non-coding repeats separately (A: coding, B: non-coding). All disease-causing CAG repeats are located in protein-coding regions except for *DMPK*, *GLS*, and *TCF4* which are in 5′UTR (Table 1). Next we tested GGC repeats. Disease-causing 5′-UTR GGC loci are long and variable (Fig. 2b) but protein-coding regions are long but show less variability (Fig. 2a). Gene names were used to indicate the disease-causing repeats because the pathogenic repeats are present only once in each gene. All known protein-coding GGC repeat diseases are located at poly-alanine tracts. This may reflect the difference in disease mechanisms of protein-coding versus 5′-UTR GGC repeats or protein-coding GGC versus CAG repeats. Next, we examined the variation and length of all intronic AAAAT repeat loci in 21 individuals, and found several highly polymorphic AAAAT repeats including disease loci (Fig. 3, Table 1).

We repeated our analysis using repeat annotations from Tandem Repeats Finder (TRF, a.k.a. simpleRepeat.txt) [26]. TRF annotates fewer repeats than tantan (Additional file 2: Figure S4A), however, the proportion of triplet repeat sequences is similar (Additional file 2: Figure S4B). Numbers of intersections between these annotations were calculated using bedtools v2.27.1 (Additional file 2: Table S2). We analyzed disease-associated CAG and GGC repeats, and observed similar results to tantan-annotated repeats (Additional file 2: Figure S5: CAG, S6: GGC, S7: AAAAT).

Next, we tested if polymorphic disease-associated tandem repeats are correlated with reported GWAS SNPs. We tested *ATXN3* and *GLS* disease-associated repeats because they are highly polymorphic among disease-associated CAG repeats. These repeats have two (rs12588287: coronary artery calcification [27], rs10143310: ALS [28]) and one (rs4853525: reticulocyte count [29]) near-by GWAS SNPs (< 10 kb) [22], respectively. Due to the limited coverage and read length, we could obtain genotypes in most but not all of the 21 cases (Additional file 1: Table S3 A, B, C). In each case, one of the two SNP alleles is significantly (*p* < 0.05, unpaired *t* test) associated with longer repeats (Additional file 2: Figure S8). Risk alleles tend to occur with shorter repeats for two SNPs: rs4853525-C and rs12588287-T. Risk allele for rs10143310 is not available [28]. This merits further investigation by genotyping a larger number of individuals.

Finally, we listed highly polymorphic repeats (IQR ≥ 5) which have very near GWAS signals (< 100 bp) from a GWAS catalog [22] (Additional file 1: Table S4). We found an interesting candidate, an intronic repeat in the *CLN8* gene: a SNP within this repeat (rs11986414) and a near-by SNP (rs4875960) are reported to be associated with severity of Gaucher syndrome [30]. It is an intriguing possibility that this repeat genuinely acts as a driver of the GWAS signals and affects the disease severity. We found that the A genotypes of these two SNPs are correlated with shorter repeat (Additional file 2: Figure S9). It would be interesting to investigate functional consequences of changing these repeats. These speculative examples need further association studies targeting near-by tandem repeats together with functional studies to elucidate the mechanistic relation to the phenotype.

## Discussion

We showed that CAG, non-coding GGC and intronic AAAAT disease-associated tandem-repeats are polymorphic and long compared to other repeats using whole genome long read sequencing data. However, coding GGC repeats did not show such variability, although the repeat lengths were longer than other repeats. It is known that poly-alanine is toxic to cells [31] and usually fewer than 10 additional alanine residues are enough to cause disease [2]. This may explain our observation that alanine-coding GGCs are less variable in the general population. In contrast, disease-associated 5′UTR GGCs are more polymorphic. One possible pathomechanism of 5′UTR GGC repeats is gene suppression as seen in fragile X syndrome [11]. Another envisioned mechanism is repeat associated non-AUG translation, which is suspected in the neurological symptoms in patients with *FMR1* premutation (more than 55 GGC repeats) [32]. The different mechanisms may reflect different variation patterns of disease-causing GGC repeats. Quintuplet AAAAT repeat loci are associated with newly-discovered types of disease, and pathomechanisms of AAAAT repeat expansions are yet unclear [15]. We also showed that there are several highly polymorphic AAAAT repeats which may be undiscovered pathogenic repeats for epilepsy.

GWAS have identified numerous genomic markers over the past fifteen years, however their functional relation to the diseases or traits is usually unclear. It is plausible that tandem repeats near those GWAS markers actually have functional relation to the traits. Interestingly, some repeat expansion disease loci may be associated with multiple diseases or traits, even when the repeat length is within the normal range [33, 34]. It is reported that polymorphic tandem repeats contribute to gene expression variation [35]. A recent study showed that tandem repeats which can alter expression of near-by genes are potential drivers of published GWAS signals [36]. Fotsing et al. listed 1380 such tandem repeats as eSTR (repeats associated with the expression of nearby genes) [36], although no Mendelian disease-causing repeats are included in eSTR, possibly because most of the known repeat diseases may not be caused by altering gene expression levels but by changing protein products. However, there may be other diseases or traits caused by altering gene expression, like Fragile X syndrome.

Importantly, among disease associated CAG repeats, the noncoding repeat in *TCF4* has high IQR. This triplet repeat was known to be highly polymorphic [37], in agreement with our result. This repeat has an association with Fuchs endothelial corneal dystrophy (FECD) (MIM#613267) [38]. Initially, GWAS showed an association of a SNP (rs613872), but later studies showed this disease has much higher association to a 43 kb-downstream CAG repeat which is in linkage disequilibrium with the GWAS SNP [6, 7]. It is intriguing to consider that further studies on polymorphic repeats may lead to the discovery of true pathogenic variants from GWAS SNPs. However, it is reported that tandem repeats with multiple genotypes are poorly tagged with SNPs [39]. Nevertheless, some repeat expansion diseases are known to be linked to certain haplotypes [40, 41], although there are repeat expansions that do not share haplotype or occur de novo [42]. We showed some examples in this study. The first example is a 5′UTR GCA repeat in the *GLS* gene, which is highly polymorphic and also listed as an eSTR [36]. Expansions (> ~ 680 repeats) are known to cause deficiency of GLS and linked to neurological disease [43]. Several lines of evidence show that an 8 kb-downstream SNP is associated with reticulocyte count (Additional file 1: Table S3 C). We showed that this SNP is correlated with repeat length. *GLS* encodes glutaminase, which catalyzes glutamine conversion to glutamate, has high activity in red blood cells (erythrocytes), and plays a role in glutathione metabolism [44, 45]. There is an intriguing possibility that this 5′UTR repeat actually acts as a driver of the GWAS signal and affects reticulocyte-erythrocyte maturation by altering the expression of *GLS* thus affecting glutathione metabolism. The next example is *ATXN3*. We found two near-by GWAS SNPs, including one associated with ALS, are significantly correlated with repeat length. Since another spinocerebellar ataxia repeat in *ATXN2* is associated with ALS, this locus is of interest. A final example is the Gaucher disease severity associated SNPs in and near the polymorphic repeat in an intron of *CLN8*. These speculative examples need further association studies targeting near-by tandem repeats together with functional studies to elucidate the mechanistic relation to the phenotype.

## Conclusion

In conclusion, our results indicate that known disease-associated coding CAG repeats, 5′UTR GGC repeats, and intronic AAAAT repeats are long and variable, but alanine-coding GGC repeats are stable (but long) among the 21 individuals. Our study is limited due to lack of a large number of healthy individuals from multiple ethnicities. Nevertheless, we provide a first example of applying long read sequencing to identify polymorphic tandem repeats. We believe further tandem-repeat surveys using a large number of individuals may provide more insights into human genomes and diseases.

## Supplementary Information

**Additional file 1.** Table S1. Data sets used in this study.Detection rates are the number of tandem repeats whose length is predicted from at least one DNA read. *3,312,291 loci. Table S2. Comparison of tantan and TRF annotated tandem repeatsNumber of intersections are counted using bedtools (https://bedtools.readthedocs.io/en/latest/). Each repeat unit was counted separately. Table S3. Phased repeat length and near-by SNPs.SNP genotype on the short and long alleles were shown in each dataset. Repeat copy number changes on both alleles were genotyped using tandem-genotypes. (A) ATXN3 repeat and (B, C) GLS repeat. SNP rs numbers are from dbSNP (https://www.ncbi.nlm.nih.gov/snp/). Table S4. Polymorphic tandem repeat and near-by GWAS signals.Highly polymorphic exonic triplet repeats (IQR ≥ 5) with near GWAS signals (< 100bp). GWAS signals were from a GWAS catalog [22].

**Additional file 2.** Figure S1. Variation of tandem repeat length (copy number) in long reads from 21 individuals. x-axis: copy number change relative to the human reference (hg38). y-axis: read number. Three different repeat types ((A) exonic CAG, (B) exonic GGC and (C) intronic AAAAT) are shown. Disease repeats: 12 CAG repeats, 14 GGC repeats, and 7 intronic AAAAT repeats. Other repeats: exonic CAG: n = 1840, exonic GGC: n = 3073, intronic AAAAT: n = 19,744. For each repeat type, we show ten sets of “other repeats” for comparison. Each set of “other repeats” is a random selection of the same number of repeats as the number of disease repeats. Figure S2. Repeat length mean and spread of each triplet repeat type. There are 10 kinds of triplet repeat, AAC, CAC, CCT, CTT, GAT, GTA, GTC, TAA CAG and GGC. The numbers of repeats are; 650 (AAC), 632 (CAC), 1862 (CCT), 737 (CTT), 430 (GAT), 47 (GTA), 73 (GTC), 682 (TAA), 1839 (CAG) and 2907 (GGC). The variation of the repeat length in 21 individuals are shown. x-axis: interquartile range (IQR), y-axis: mean repeat length (bp). In merged boxplots, ranges are the 25th and 75th percentiles, dots are outliers and lines in boxes are median. Figure S3. Variability of exonic repeats. Number of exonic tandem repeats (A) and IQR (B) of each unit are shown. Shorter-unit repeats have more variation. Dots represent outliers. Boxplot ranges are the 25th and 75th percentiles. Lines in boxes are median. Most of the IQRs from repeats whose length are more than six are zero. Figure S4. Comparison of tantan and TRF annotated tandem repeats (A) There are more tantan annotated tandem-repeats than TRF-annotated repeats, however, the distribution of the number of the loci has similar tendency. x-axis: length of repeat unit, y-axis: number of loci. (B) Proportions of triplet repeat sequences are similar between tantan and TRF annotation. Figure S5. Variability of CAG repeats using TRF-annotated repeats.Variation (IQR) and length of repeats with disease-associated sequences. Coding CAG repeats (A), and non-coding exonic CAG repeats (B). x-axis: IQR, y-axis: mean repeat length (bp). n provides the numbers of repeat loci. In merged boxplots on the right and upper, ranges are the 25th and 75th percentiles, dots are outliers and lines in boxes are median. Figure S6. Variability of GGC repeats using TRF-annotated repeats.Variation (IQR) and length of repeats with disease-associated sequences. Coding GGC repeats (A), and non-coding exonic GGC repeats (B). x-axis: IQR, y-axis: mean repeat length (bp). n provides the numbers of repeat loci. In merged boxplots on the right and upper, ranges are the 25th and 75th percentiles, dots are outliers and lines in boxes are median. Figure S7. Variability of AAAAT repeats using TRF-annotated repeats.Variation (IQR) and length of repeats with disease-associated intronic AAAAT sequences. x-axis: IQR, y-axis: mean repeat length (bp). n provides the numbers of repeat loci. In merged boxplots on the right and upper, ranges are the 25th and 75th percentiles, dots are outliers and lines in boxes are median. Figure S8. Repeat length correlates with near-by SNPs. (A) Distribution of tandem repeat length (copy number) in combined long reads of GLS and ATXN3 disease-associated repeats from 21 individuals. x-axis: copy number change relative to the human reference (hg38). y-axis: read count. (B) Three GWAS reported SNPs were near the GLS and ATXN3 repeats. Repeat lengths of each genotype were compared using unpaired t test. *P* values are shown. Figure S9. Gaucher disease severity modifying SNPs correlate with repeat length.(A) Distribution of tandem repeat length (copy number) in combined long reads of CLN8 from 21 individuals. Note that there is bimodal distribution, with peaks around zero and − 13 copy number changes. x-axis: copy number change relative to the human reference (hg38). y-axis: read count. (B) One GWAS reported SNP rs11986414 is inside and another SNP rs4875960 is near this repeat. In both SNPs, genotype A tends to have larger repeat length.

## Data Availability

PromethION WGS sequence data is available from DDBJ (DRA009852). Other public data were downloaded from NCBI or Human PanGenome Project (https://github.com/human-pangenomics/hpgp-data) under accession numbers described in Table S1. SNP identifiers were used under accession numbers from dbSNP (https://www.ncbi.nlm.nih.gov/snp/).

## Abbreviations

GWAS: Genome-wide association studies
SNP: Single nucleotide polymorphism
IQR: Interquartile range
FECD: Fuchs endothelial corneal dystrophy
