# Supplementary material for: Genome-wide survey of tandem repeats by nanopore sequencing shows that disease-associated repeats are more polymorphic in the general population
Source: BMC Med Genomics. 2021 Jan 7;14:17. doi: 10.1186/s12920-020-00853-3 (PMC7791882; doi:10.1186/s12920-020-00853-3)

Supplementary Figure 1

A

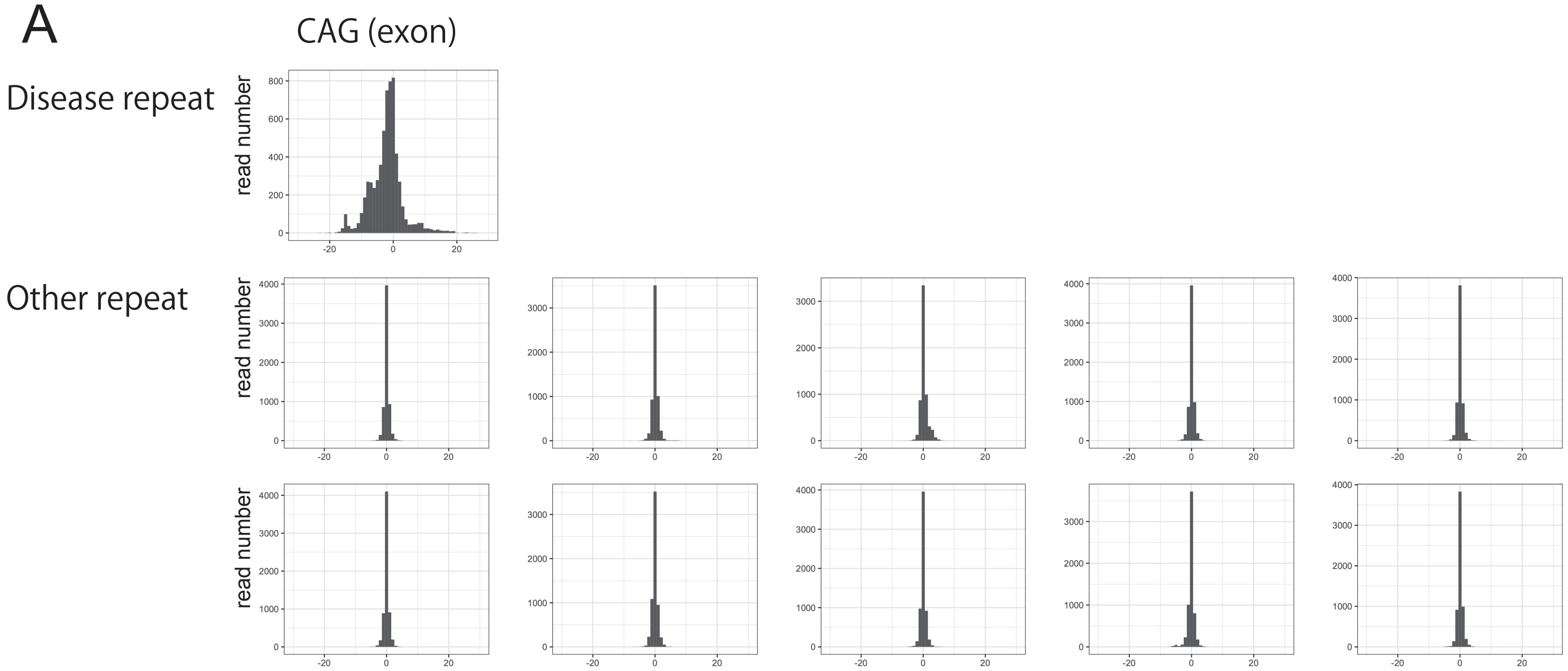

x-axis= copy number change relatie to the human reference

B

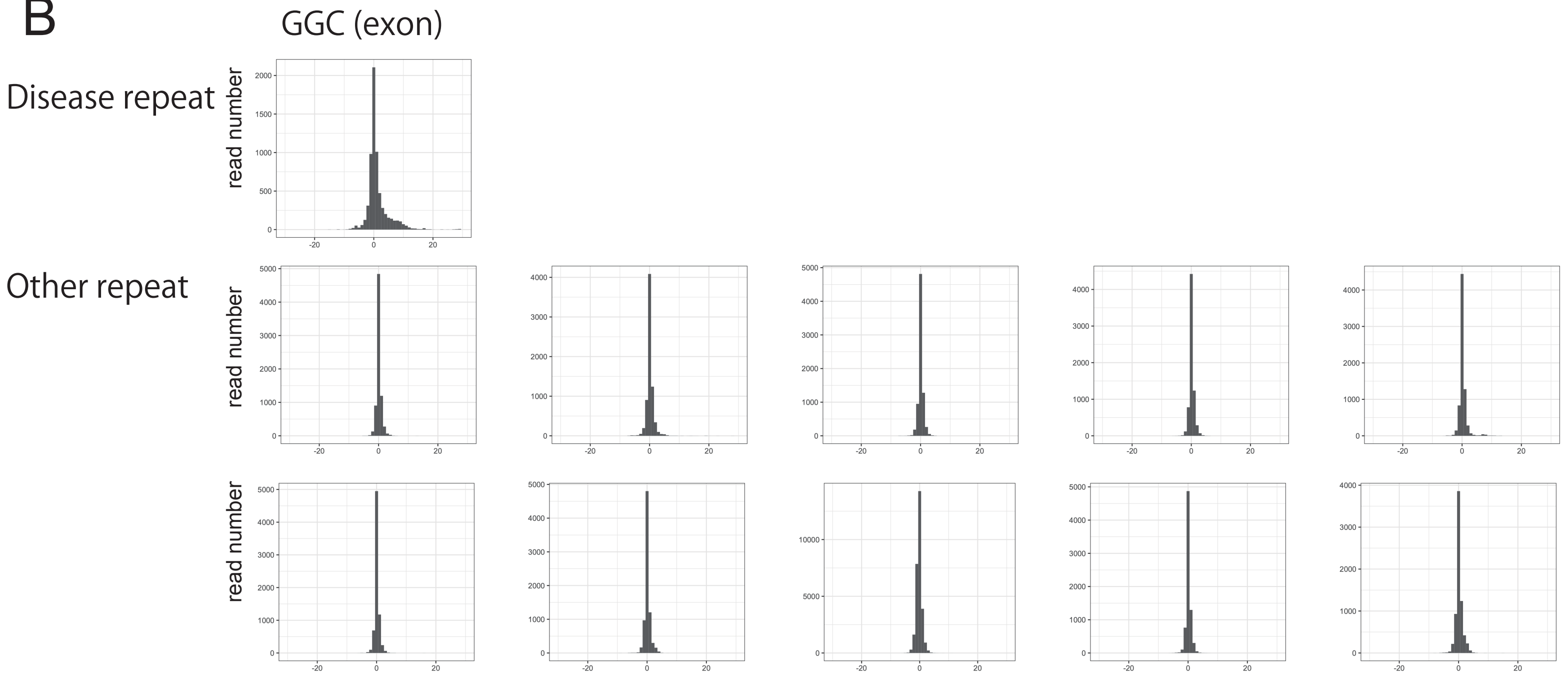

x-axis= copy number change relatie to the human reference

C

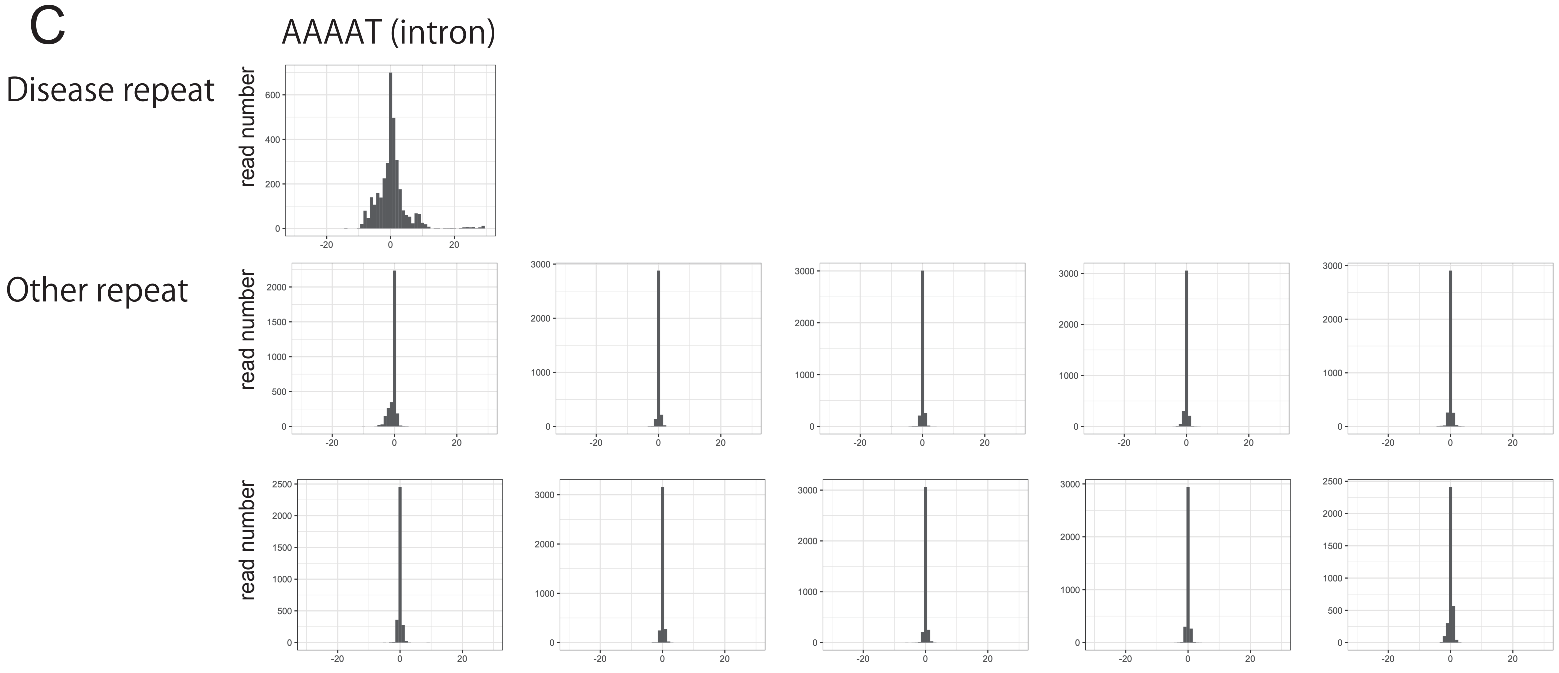

x-axis= copy number change relatie to the human reference

Supplementary Figure S2

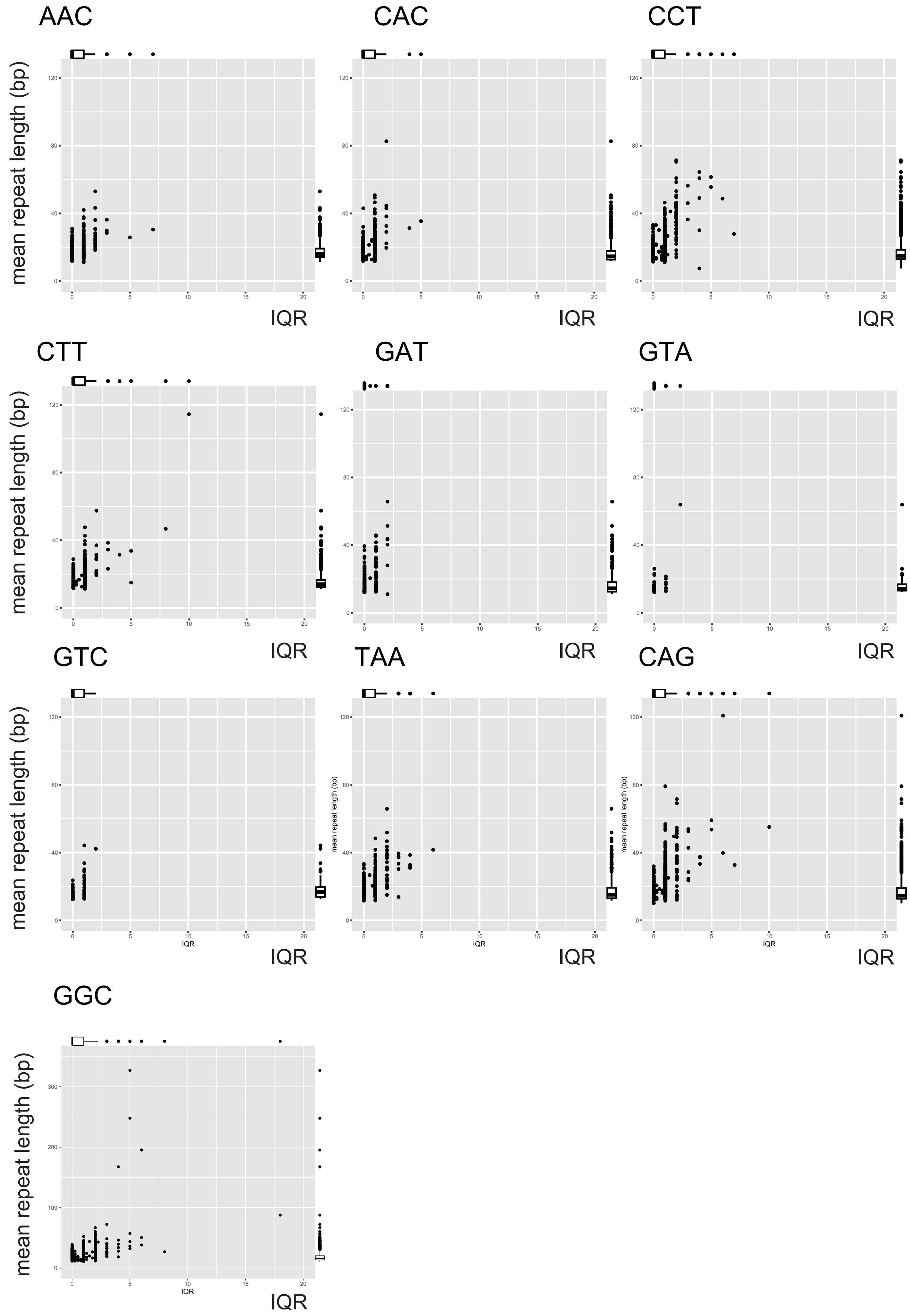

Supplementary Figure S3

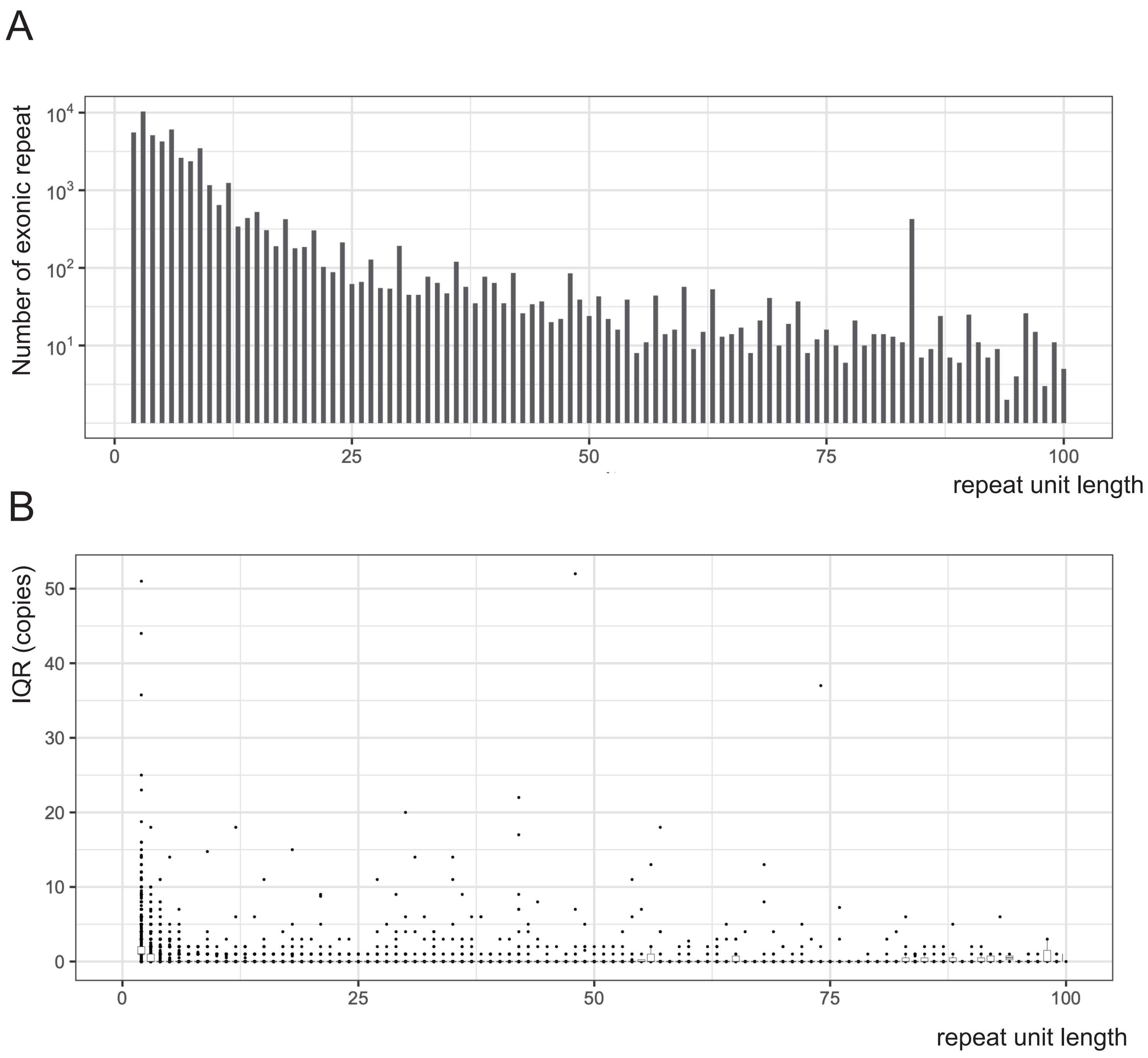

Supplementary Figure S4

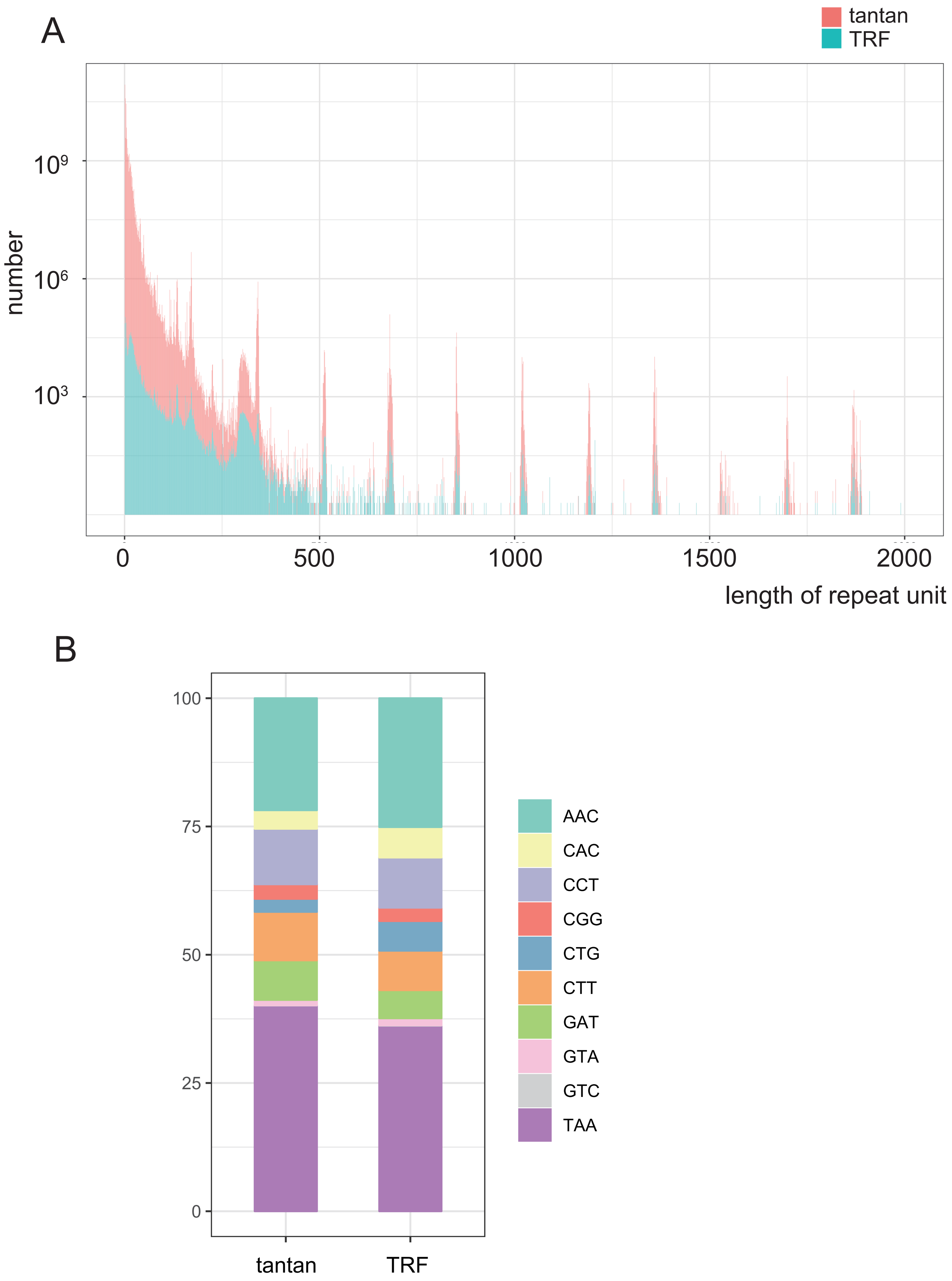

Supplementary Figure S5

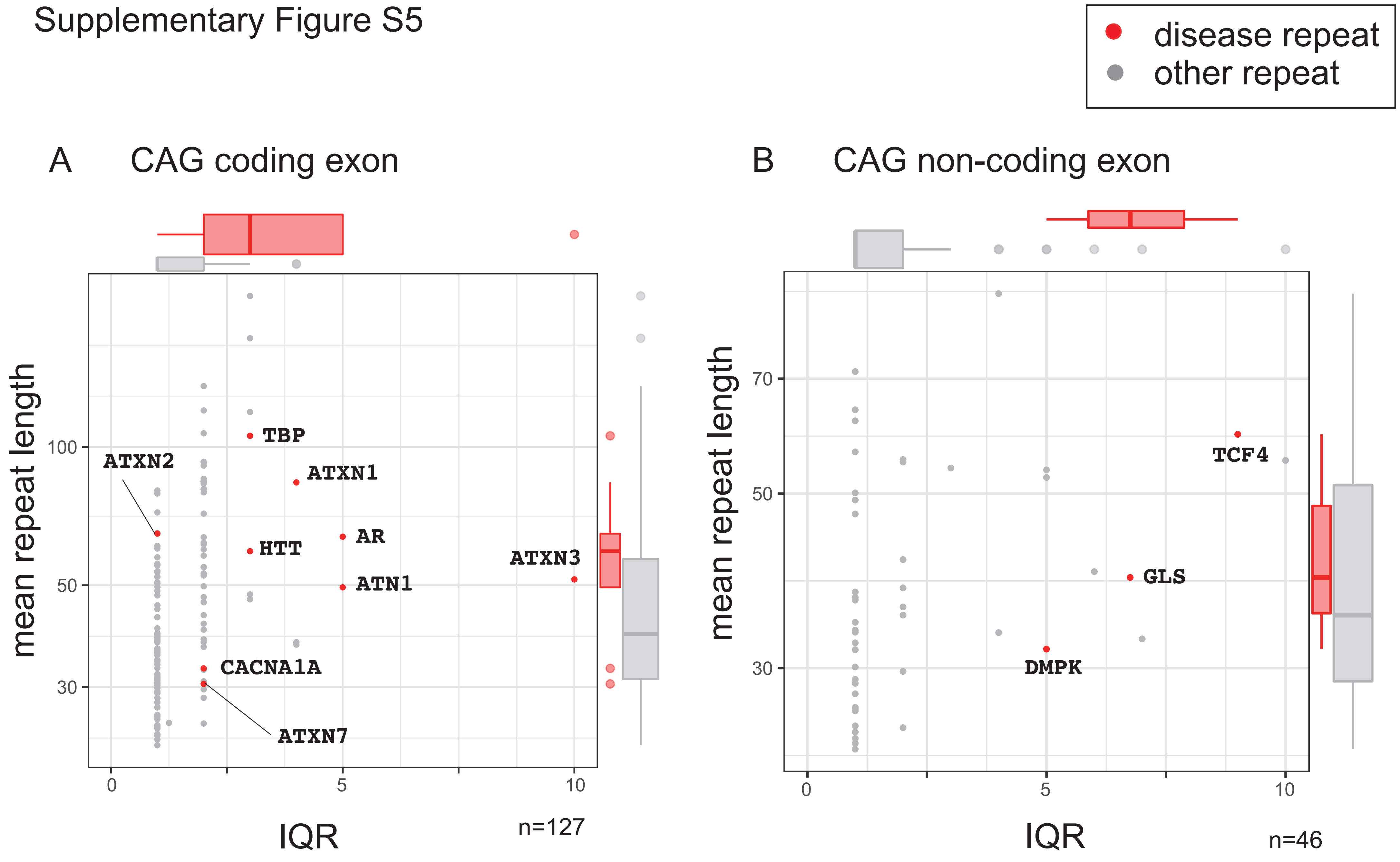

Supplementary Figure S6

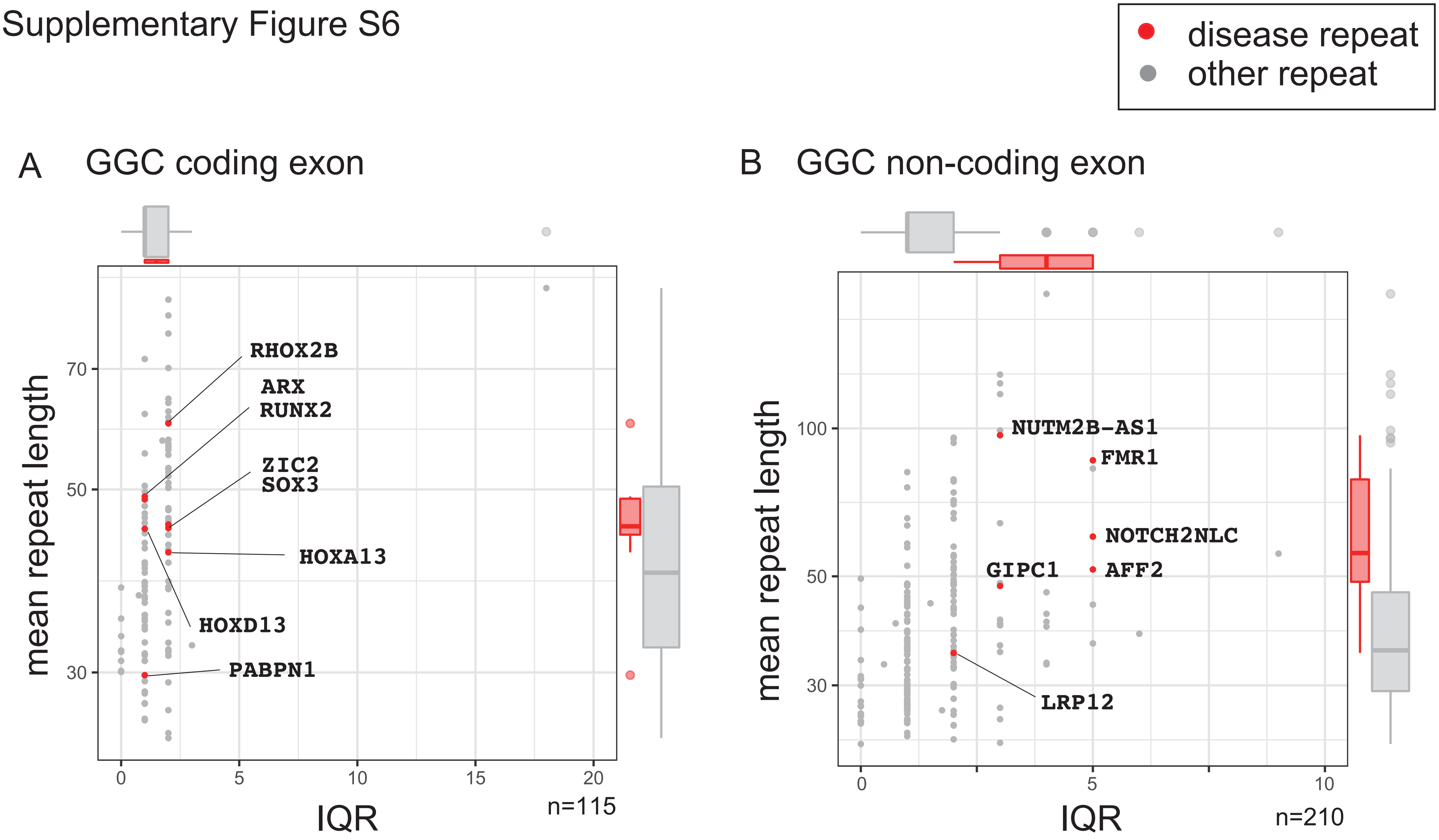

Supplementary Figure S7

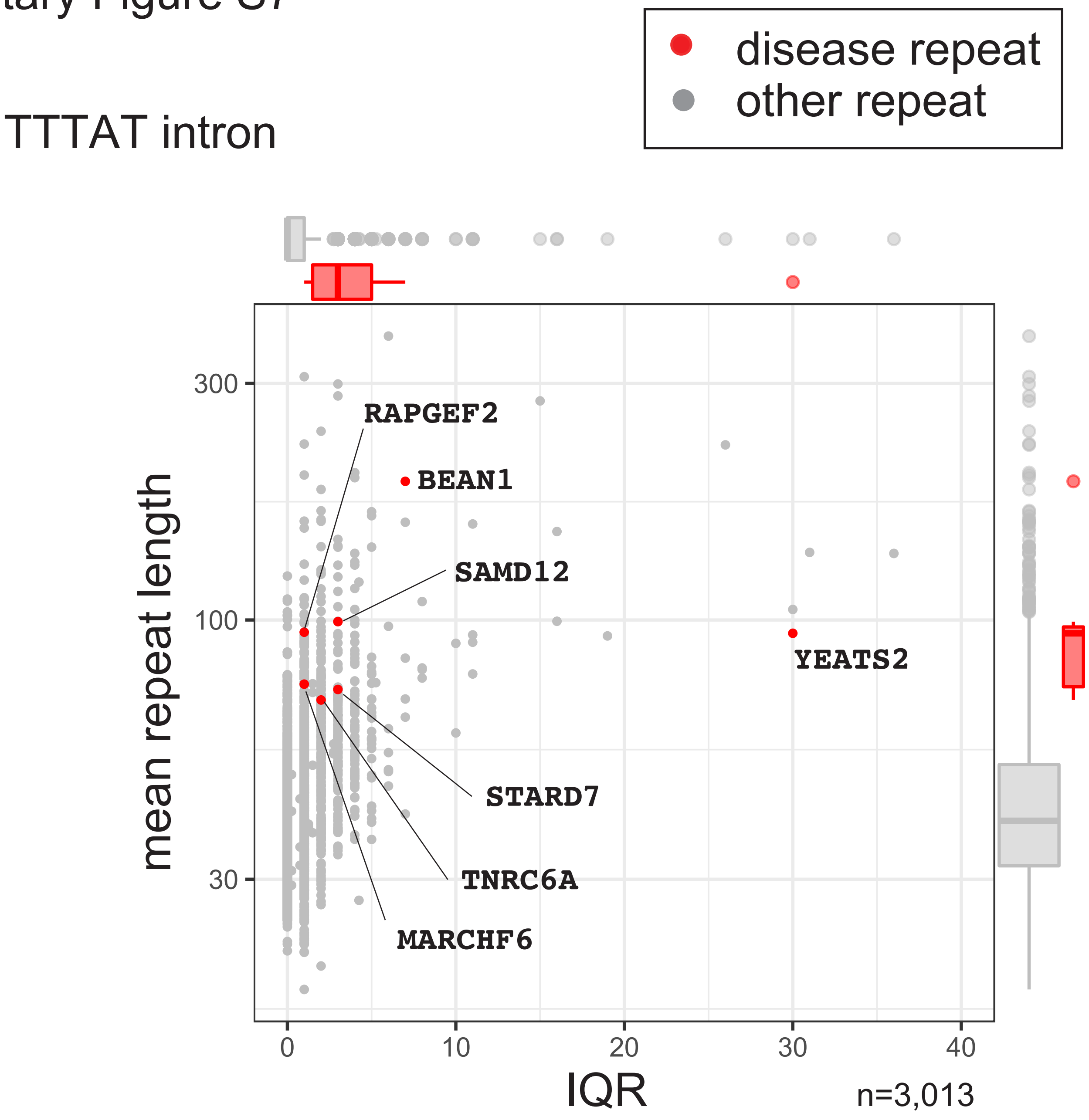

Supplementary Figure S8

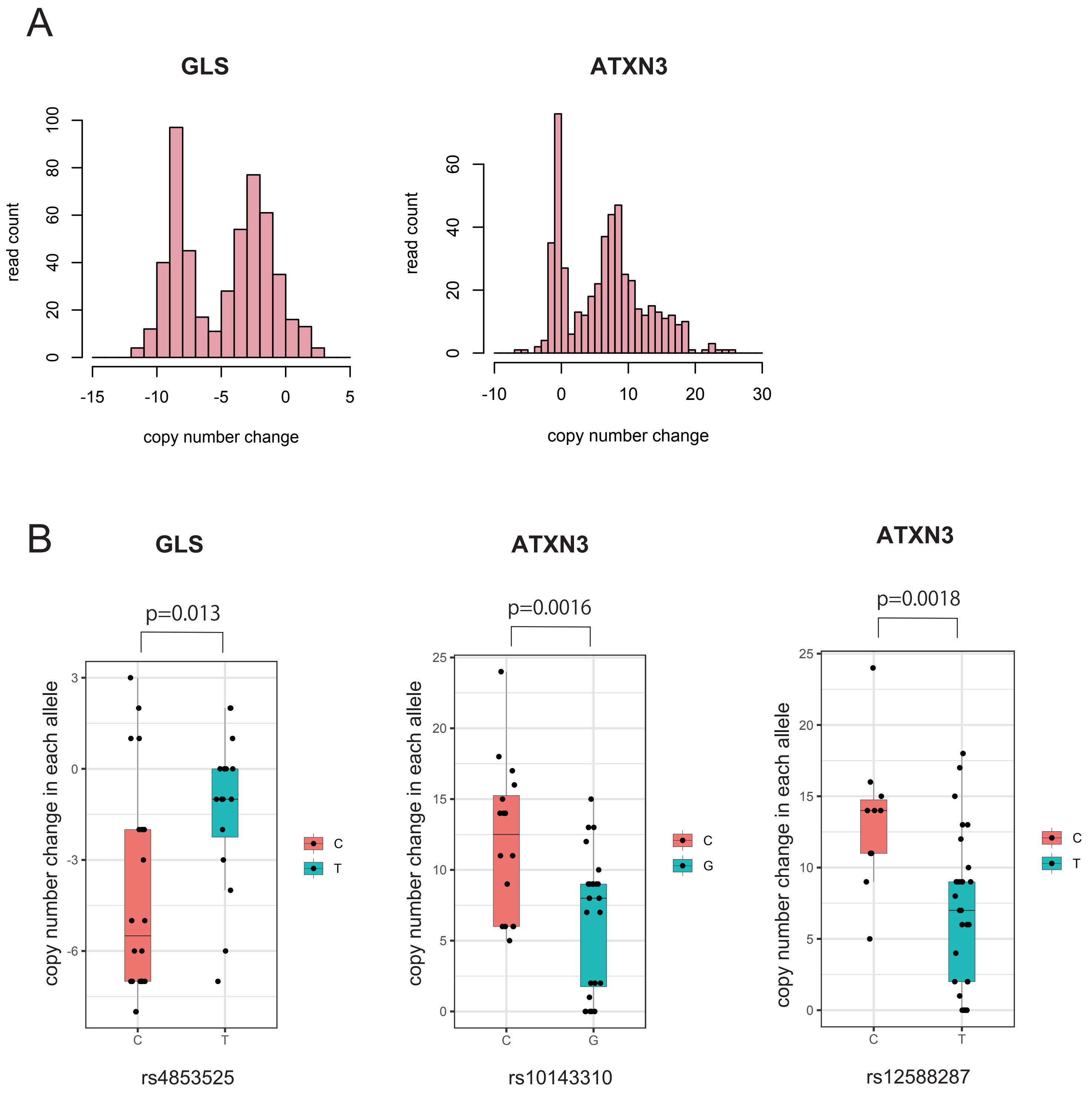

Supplementary Figure S9

A

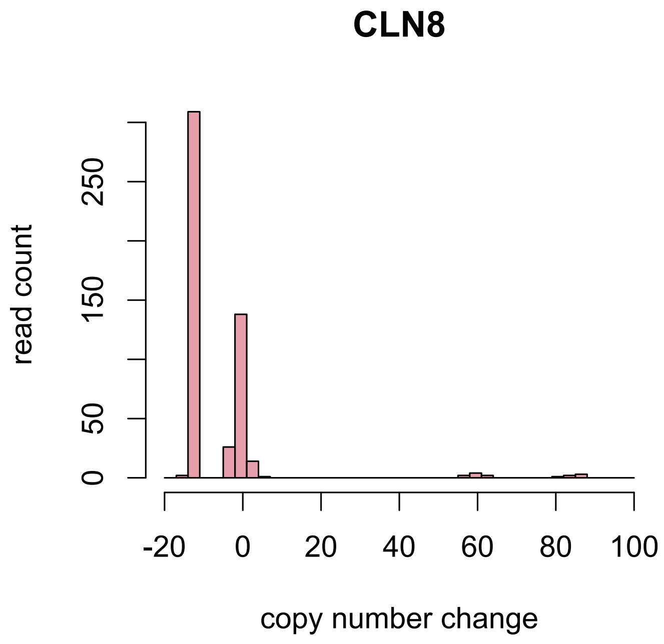

B

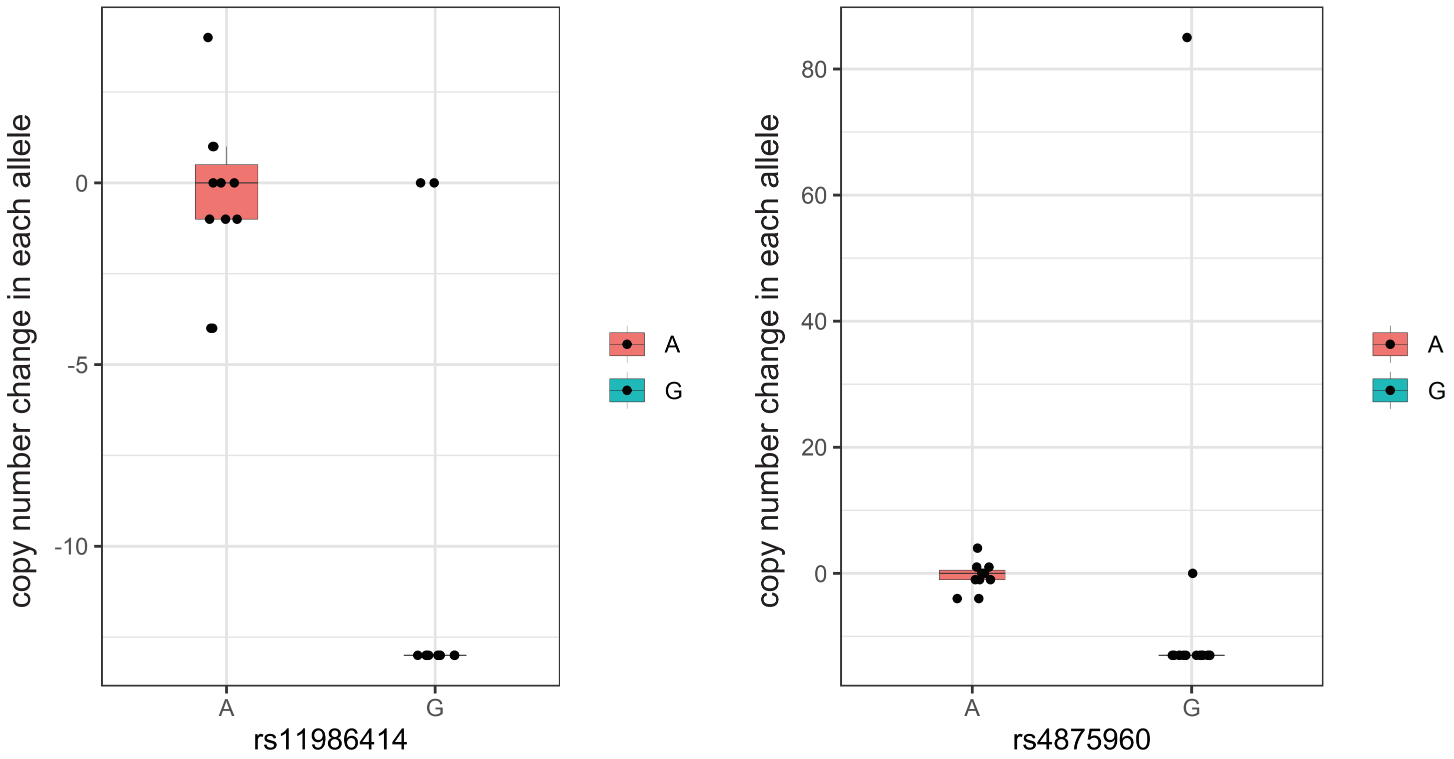

Supplement: Supplementary file 2 — Additional file 2. Figure S1. Variation of tandem repeat length (copy number) in long reads from 21 individuals. x-axis: copy number change relative to the human reference (hg38). y-axis: read number. Three different repeat types ((A) exonic CAG, (B) exonic GGC and (C) intronic AAAAT) are shown. Disease repeats: 12 CAG repeats, 14 GGC repeats, and 7 intronic AAAAT repeats. Other repeats: exonic CAG: n = 1840, exonic GGC: n = 3073, intronic AAAAT: n = 19,744. For each repeat type, we show ten sets of “other repeats” for comparison. Each set of “other repeats” is a random selection of the same number of repeats as the number of disease repeats. Figure S2. Repeat length mean and spread of each triplet repeat type. There are 10 kinds of triplet repeat, AAC, CAC, CCT, CTT, GAT, GTA, GTC, TAA CAG and GGC. The numbers of repeats are; 650 (AAC), 632 (CAC), 1862 (CCT), 737 (CTT), 430 (GAT), 47 (GTA), 73 (GTC), 682 (TAA), 1839 (CAG) and 2907 (GGC). The variation of the repeat length in 21 individuals are shown. x-axis: interquartile range (IQR), y-axis: mean repeat length (bp). In merged boxplots, ranges are the 25th and 75th percentiles, dots are outliers and lines in boxes are median. Figure S3. Variability of exonic repeats. Number of exonic tandem repeats (A) and IQR (B) of each unit are shown. Shorter-unit repeats have more variation. Dots represent outliers. Boxplot ranges are the 25th and 75th percentiles. Lines in boxes are median. Most of the IQRs from repeats whose length are more than six are zero. Figure S4. Comparison of tantan and TRF annotated tandem repeats (A) There are more tantan annotated tandem-repeats than TRF-annotated repeats, however, the distribution of the number of the loci has similar tendency. x-axis: length of repeat unit, y-axis: number of loci. (B) Proportions of triplet repeat sequences are similar between tantan and TRF annotation. Figure S5. Variability of CAG repeats using TRF-annotated repeats.Variation (IQR) and length of repeats with di [file 12920_2020_853_MOESM2_ESM.pdf]
